# Proniosomes derived niosomes: recent advancements in drug delivery and targeting

**DOI:** 10.1080/10717544.2017.1384520

**Published:** 2017-11-12

**Authors:** Maryam Khatoon, Kifayat Ullah Shah, Fakhar Ud Din, Shefaat Ullah Shah, Asim Ur Rehman, Naz Dilawar, Ahmad Nawaz Khan

**Affiliations:** a Department of Pharmacy, Quaid-e-Azam University, Islamabad, Pakistan;; b Department of Pharmaceutics, Faculty of Pharmacy, Gomal University, D.I. Khan, Pakistan;; c School of Chemical and materials Engineering, National University of Sciences and Technology, Islamabad, Pakistan

**Keywords:** Proniosomes, hydration, niosomes, drug delivery, targeting, advancements, transdermal

## Abstract

Vesicular drug delivery systems have gained wide attention in the field of nanotechnology. Among them proniosomes become the superior over other vesicular carriers. Proniosomes are dry formulations of water soluble nonionic surfactant coated carrier system which immediately forms niosomes upon hydration. They have the capability to overcome the instability problems associated with niosomes and liposomes and have the potential to improve solubility, bioavailability, and absorption of various drugs. Furthermore, they offer versatile drug delivery concept for enormous number of hydrophilic and hydrophobic drugs. They have the potential to deliver drugs effectively through different routes at specific site of action to achieve controlled release action and reduce toxic effects associated with drugs. This review discusses the general preparation techniques of proniosomes and mainly focus on the applications of proniosomes in drug delivery and targeting. Moreover, this review demonstrates critical appraisal of the literature for proniosomes. Additionally, this review extensively explains the potential of proniosomes in delivering drugs via different routes, such as oral, parenteral, dermal and transdermal, ocular, oral mucosal, vaginal, pulmonary, and intranasal. Finally, the comparison of proniosomes with niosomes manifests the clear distinction between them. Moreover, proniosomes need to be explored for proteins and peptide delivery and in the field of nutraceuticals and develop pilot plant scale up studies to investigate them in industrial set up.

## Introduction

Novel vesicular drug delivery systems have made great progress in the field of nanotechnology. As these systems have a potential to carry a variety of drugs and have been widely used for various purposes, such as drug targeting, controlled release, and permeation enhancement of the drugs (Akhilesh et al., 2011; Mir et al., 2017). These systems are also valuable in evading various drawbacks associated with conventional dosage forms like low aqueous solubility, poor bioavailability, poor membrane permeability, variable plasma concentration, undesirable effects, poor patient compliance, and finally poor patient efficacy (Bochot & Fattal, 2012; Zaki Ahmad et al., 2014; Song et al., 2015). From the last few decades, with various novel drug delivery system approaches including solid lipid nanoparticles, dual reverse thermosensitive systems, complexation, electro spraying, solid dispersions, co-solvency and nanosizing (nanoemulsion, nanosuspension, nanoparticles, and nanocrystals) had been proposed to resolve these issues (Ud Din et al., 2015a,b, 2017; Rashid et al., 2015a, 2015b; Ahmad et al., 2016; Mustapha et al., 2016).

However, too much emphasis was made on vesicular drug carriers, such as liposomes or niosomes for demonstrating supremacy over conventional dosage forms which involves the encapsulation of drug within vesicles to achieve prolonged effect of drugs and minimize toxic effects by drug targeting (Aburahma & Abdelbary, 2012). Both these systems act as a drug reservoir, can carry both hydrophilic and hydrophobic drug by encapsulation and partitioning in hydrophobic domains and exist in forms of unilamellar or multilamellar spherical structures enclosed by a membrane (Kamel et al., 2013). Among all the vesicular systems phospholipid vesicles (liposomes) had attracted greatly by the formulation scientists due to their distinctive potential to enclose a wide variety of substances and drugs and to encapsulate both hydrophilic and lipophilic compounds (Mahale et al., 2012; Pham et al., 2012; Hua & Wu, 2013). Despite these advantages, there are some physical and chemical instabilities associated with liposomes like aggregation, fusion or leakage upon storage, degradation of phospholipids by hydrolysis, and oxidation in a dispersed aqueous system that makes them unfit for oral administration (Hu & Rhodes, 1999; Chaw & Ah Kim, 2013; Yasam et al., 2014; Ahmad et al., 2017).

To overcome the physical instabilities associated with liposomes two novel concepts were proposed; to develop proliposomes which are dry, free-flowing granular powder that can be hydrated immediately before use and to develop nonionic surfactant vesicles containing nonionic surfactants instead of phospholipids. Proliposomes are more stable for longer duration without significant changes during sterilization and storage (Janga et al., 2012). However, the problem of degradation of phospholipids by oxidation was still there. Afterwards, to avoid all these drawbacks an alternative approach was emerged involving preparation of liposome like vesicles called niosomes. Niosomes or nonionic surfactant carriers are alternative and identical to liposomes in physical structure. These are aqueous dispersions of lipid vesicles made from safe and biocompatible nonionic surfactants with or without cholesterol in which hydrophilic drugs can be incorporated in an inner core and lipophilic drugs in an outer lipid bilayer (Elhissi et al., 2013; Tu et al., 2014; Shukr & Eltablawy, 2015). Niosomes serves as promising drug carriers as these are able to circumvent the limitations associated with liposomes, because these contain surfactants which are easily derivatized and gives higher versatility to the vesicular structure (Marianecci et al., 2014).

Niosomal formulations could improve the solubility of some poorly soluble drugs and bioavailability as shown for acyclovir and griseofulvin and control the drug delivery with good chemical stability during storage, e.g. the stability of peptide drugs can be significantly increased by encapsulation (Rajera et al., 2011). Furthermore, low cost, more stability, entrapping of more substances, ease of handling, ease of formulation and storage, less prone to oxidation and availability of prepared materials in pure form exploit them a propitious drug delivery system, and superior to liposomes (Paecharoenchai et al., 2013; Zaki Ahmad et al., 2014). Niosomes also undergo fusion, aggregation, sedimentation, leakage of entrapped drugs on storage, and loss of vesicular integrity thus, limiting their shelf life (El-Laithy et al., 2011; Shaker et al., 2013; El Maghraby et al., 2015; Madan et al., 2016).

**Figure 1.**
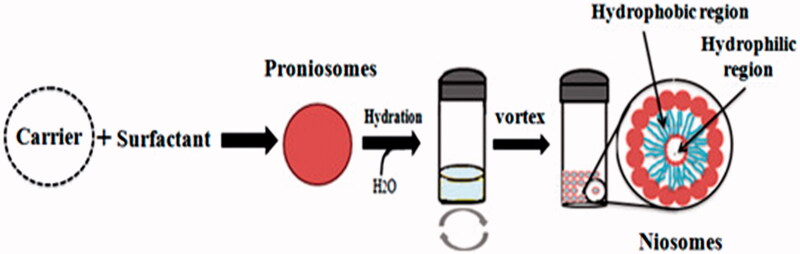
Hydration of proniosomes into niosomes and hydrophilic and hydrophobic regions of niosomes.

To overcome all the defects related to other vesicular drug delivery systems, there was a need of hour to design such a system that is more physically and chemically stable. A feasible and the latest approach to formulate stable niosomes is the provesicular carrier system, i.e. proniosomes. Proniosomes are either anhydrous free flowing formulations or liquid crystals with jelly like consistency of water-soluble carrier coated with the suitable noisome-forming surfactants. They can easily be reconstituted with aqueous phase before administration or hydrated in body compartments to form niosomal vesicles (Figure 1) and these proniosomes-derived niosomes are better than conventional niosomes (Rahman et al., 2015; Shehata et al., 2015). This proniosomal technology can diminish physical and chemical instabilities associated with niosomes by avoiding their storage in aqueous medium (Najlah et al., 2015). Proniosomes offer a potential vesicular drug delivery concept and may be a promising transporter for lipophilic medications (Mehta et al., 2016). Both phospholipids and nonionic surfactants in proniosomes can act as penetration enhancers (Ammar et al., 2011). The ratio between the nonionic surfactant and cholesterol could affect both the release characteristics and the entrapment efficiency of the incorporated drugs (Zidan & Mokhtar, 2011). These vesicles can encapsulate both hydrophilic and hydrophobic drugs (Figure 1) and do not require specific storage conditions. Proniosomes, also called ‘dry niosomes’, offers additional convenience of transportation; distribution, storage, and dosing that make them an efficient delivery system with potential for use with a wide range of active compounds (El-Laithy et al., 2011; Rahman et al., 2015; Shehata et al., 2015). The encapsulation of drug in the proniosomal vesicular structure maintains their systemic circulation, provides controlled release, enhances penetration in the targeted areas and reduces the toxic effects (Akhilesh et al., 2012).

This review will predominantly focus on functionality of proniosomes in the field of drug delivery and targeting. Particularly, the potential of proniosomes in delivering drugs via different routes, such as oral, parenteral, topical and transdermal, ocular, vaginal, mucosal, pulmonary, and intranasal. Similarly, a critical preview is also provided to explore the problems associated with the proniosomes, their functionality and efficacy in the above discussed fields for their future excellency in the fields of drug delivery and targeting.

## Methods of preparation

**Figure 2.**
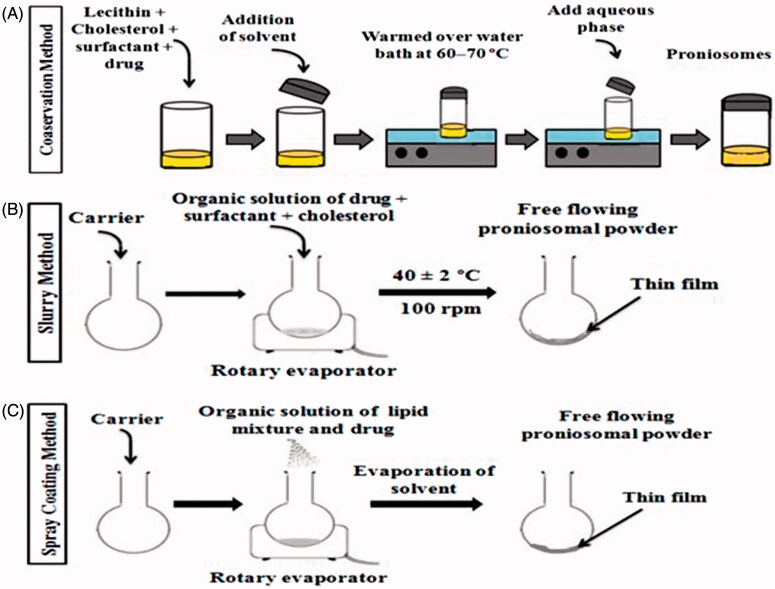
Methods of preparation of proniosomes (A) Coacervation phase separation method (B) Slurry method (C) Spraying method.

Different methods are reported for the preparation of proniosomes, such as coacervation phase separation method, slurry method and the spraying of nonionic surfactant on water-soluble carrier particles. The description of preparation methods and their sequential steps are explained in Table 1 and Figure 2(A–C).

**Table 1.** Description of preparation methods, their principle and type of formulation formed.

Preparation method	Principle	Formulation type	References
Coacervation phase separation method	Mixing of lipids, surfactant and drug with the solvent followed by the warming of mixture over water bath at 60–70 °C until translucent dispersion is obtained.	Proniosomal gel	Yasam et al. (2014); Ammar et al. (2011); Ahmad et al. (2017)
Slurry method	Slurry preparation by using organic solution of cholesterol, surfactant, drug, and then poured onto carrier material. Evaporate the solvent in rotary evaporator to form free flowing proniosomes.	Proniosomal powder	Mujoriya & Bodla (2011); Walve et al. (2011)
Spray coating method	Successive spraying of organic solution of cholesterol, surfactant, and drug onto carrier material containing in round bottom flask attached to rotary evaporator.	Proniosomal powder	Nasr (2010)

### Proniosomes in drug delivery and targeting

Proniosomes are successful drug delivery system and have acquired great attention by the researchers as a versatile vesicular drug carrier and targeting agent since 1980’s (Kumar & Rai, 2012). To enlighten the usefulness of proniosomes in drug delivery and targeting various publications will be discussed in this section that evident the applications of proniosomes in effective delivery of wide range of therapeutic agents through different routes, such as oral, parenteral, dermal, transdermal, ocular, pulmonary, vaginal, and mucosal route (Table 2).

**Table 2.** Outline of drug delivery applications of proniosomes through different routes, composition and their *in vitro*/*in vivo* effects.

Route	Drug	Composition	*In vitro*/*in vivo* effects	References
Oral	Vinpocetine	Span 60/sorbitol/cholesterol	Improve oral bioavailability and GI absorption	Song et al. (2015)
	Candesartan cilexetil	Span 60/maltodextrin/cholesterol	Improve oral bioavailability	Yuksel et al. (2016)
	Acemetacin	Span 60/maltodextrin/cholesterol/stearylamine	Enhance pharmacokinetic properties and anti-inflammatory effects	Shehata et al. (2015)
	Pioglitazone	Span 60/maltodextrin/cholesterol	Improve hypoglycemic effects by controlled release of drug	Shukr & Eltablawy (2015)
	Nateglinide	Span 60/maltodextrin/cholesterol	Improve oral bioavailability	Sahoo et al. (2014)
	Doxycycline hydrochloride and metronidazole	Span 60/maltodextrin/cholesterol	Improve combination therapy and patient compliance	Gad et al. (2014)
	isradipine	Span 40: Span 60/cholesterol/dicetyl phosphate	Improve oral bioavailability and gastrointestinal (GI) absorption	Veerareddy & Bobbala (2013)
	Diphenyl dimethyl bicarboxylate	Tween 80/sorbitol/cholesterol/stearylamine	Enhance dissolution and hepatocurative activity	Aburahma & Abdelbary (2012)
	Valsartan	Span 60/maltodextrin/cholesterol	Improve oral bioavailability and enhanced permeation	Gurrapu et al. (2012)
Parenteral	Flurbiprofen	Span 80: Span 20/Sorbitol/cholesterol	Sustained anti-inflammatory activity and reduce dosing frequency	Verma et al. (2016)
Dermal	Boswellic acid	Span 40/cholesterol/soya lecithin	Improve bioavailability, absorption and release kinetics	Mehta et al. (2016)
	Tretinoin	Span 60/sorbitol/cholesterol	Enhance efficacy and reduce side effects	Rahman et al. (2015)
Transdermal	Tenoxicam	Tween 20/cholesterol	Improve patient compliance and drug safety	Ammar et al. (2011)
	Lornoxicam	Lutrol F68/cholesterol/lecithin	Improve transdermal delivery	Madan et al. (2016)
	Mefenamic acid	Span 80/cholesterol/soya lecithin	Improve transdermal delivery and anti-inflammatory activity	Wen et al. (2014)
	Lacidipine	Cremophor RH 40/cholesterol/soya lecithin	Improve transdermal delivery, absorption and permeation	Soliman et al. (2016)
	Simvastatin	Tween 20/lecithin	Enhance bioavailability and hypocholesterolemic effect	Shaker et al. (2013)
	Vinpocetine	Sugar ester/cholesterol/lecithin	Improve absorption and penetration	El-Laithy et al. (2011)
	Flurbiprofen	Cholesterol	Improve solubility and permeation	Zidan & Mokhtar (2011)
	Oxybutynin chloride	Span 20: Span 60/cholesterol/soya lecithin	Enhance drug permeation and therapeutic effect	Rajabalaya et al. (2016)
	Tolterodine tartrate	Span 20: Span 60/cholesterol/lecithin	Reduce side effects and effective management of overactive bladder	Rajabalaya et al. (2016)
	Risperidone	Span 60/cholesterol/phospholipid G 90	Increase skin permeability and bioavailability	Imam et al. (2015)
Oral mucosal and dental	Lornoxicam	Span 60/cholesterol/lecithin	Improve patient compliance and reduce gastrointestinal (GI) side effects	Abdelbary & Aburahma (2015)
	Benzocaine	Span 60/cholesterol	Improve local anesthesia by controlled release	El-Alim et al. (2014)
Ocular	Lomefloxacin HCl	Span60:Tween60/cholesterol/	Improve ocular bioavailability and prolong corneal retention	Khalil et al. (2016)
	Tacrolimus	Poloxamer 188/cholesterol/Lecithin	Delay corneal allograft rejection and prolong survival time of corneal allografts	Li et al. (2014)
Vaginal	Terconazole	Span 60: Brij76/Cholesterol/lecithin	Enhance mucoadhesive properties	Abdou & Ahmed (2016)
Pulmonary	Beclomethasone dipropionate	Span 60/cholesterol	High drug output and fine particle fraction (FPF)	Elhissi et al. (2013)
	Cromolyn sodium	Sucrose stearate/cholesterol/stearylamine	Controlled drug release and improve aerosolization	Abd-Elbary et al. (2008)

#### Oral delivery

Oral delivery is the most commonly used and favorable route of drug administration. Despite, due to many problems associated with drugs administered through oral route, such as low stability in gastrointestinal tract, pre-systemic degradation of drugs by acidic or enzymatic action, and low permeability through intestinal epithelium, many drugs are administered parenterally to augment their bioavailability (Banerjee & Onyuksel, 2012). Therefore, different nanocarriers are employed to enhance the drug absorption through oral route, such as liposomes (Caddeo et al., 2017), niosomes (Khan et al., 2016), lipid nanoparticles (Kraft et al., 2014), micelles (Chen et al., 2013), gold nanoparticles (Chen et al., 2014a), and quantum dots (Chen et al., 2014b). Proniosomes have been extensively investigated as potential oral drug delivery system. Several studies have been reported which prove the utility of oral proniosomal powders in providing the enhanced solubility and bioavailability for poorly soluble drugs (Nasr, 2010).

Song et al. (2015) showed that proniosomes are promising carrier in industrial production by formulating free-flowing and stable proniosomes of poorly water-soluble drug vinpocetine to augment its oral bioavailability and gastrointestinal absorption. The results of the study unveiled that the increased bioavailability of vinpocetine encapsulated in proniosomes is due to the following two causes. First, the niosomes formed after hydration of proniosomes in gastrointestinal tract have bioadhesive property to adhere to the gastric wall proceeded by endocytosis. Second, niosomes possibly enhance the lymphatic transport that was capable of avoiding first pass effect and ultimately leads to higher bioavailability. Another cause is the use of surfactant, i.e. span 60. Surfactants have the capability to open tight junctions of intestinal epithelial wall temporarily by provoking ultra-structural changes and thereby, enhance intestinal epithelial permeability (Song et al., 2015). Similarly, the hepatocurative activity of diphenyl dimethyl bicarboxylate (DDB) is enhanced by designing provesicular system of DDB using sorbitol as a carrier that increases its solubility and oral bioavailability (Aburahma & Abdelbary, 2012). Likewise, Veerareddy & Bobbala (2013) developed the proniosomes of isradipine to enhance its oral bioavailability and the bioavailability was enhanced 2.3 fold than the control (oral suspension) (Veerareddy & Bobbala, 2013). Maltodextrin based free flowing proniosomes of nateglinide for diabetes was produced by (Sahoo et al., 2014). The oral bioavailability and absorption of nateglinide was improved by encapsulating it in proniosomes. The study observed considerably higher plasma concentration of nateglinide by this system as compared to pure drug in rabbits (Sahoo et al., 2014). Hence, it is confirmed that proniosomes are convenient carrier for oral delivery of certain poorly water-soluble drugs and have the potential to form stable niosomal carrier systems as the proniosomes are stable at 4 °C.

Furthermore, Shukr & Eltablawy (2015) formulated proniosomes by successfully encapsulating Pioglitazone HCl to assess the viability of proniosomes as stable vesicular carrier system. The *in vivo* hypoglycemic effects of pioglitazone enclosed in proniosomes revealed excessive percent decrease in blood glucose levels in diabetic rats. These proniosomes have adequate powder flow properties and acceptable for further processing into tablets or capsules (Shukr & Eltablawy, 2015). Proniosomes have vast potential of improving oral delivery of certain lipophilic, such as anti-cancer agents and amphiphilic drugs as shown by (Gurrapu et al., 2012). The dissolution and gastric absorption of valsartan was enhanced by maltodextrin-based proniosomes by developing dry proniosome powders using equal ratios of span 60 and cholesterol. Results of *in vitro* dissolution study carried out in simulated gastric and intestinal fluid revealed improved dissolution with respect to pure drug. *Ex vivo* studies showed enhanced permeation of valsartan from proniosomes across rat intestinal membrane (Gurrapu et al., 2012). The oral bioavailability of candesartan cilexetil was also improved by formulating provesicular system. The dry granular form of proniosomes can be fabricated as tablets, capsules or solutions to prepare prior usage for oral administration. Proniosomes in tablet form are propitious drug delivery carrier in enhancing the pharmacological effects of certain drugs by controlling the release and reducing the dosing frequency. Proniosomal acemetacin tablets were successfully prepared by direct compression of free flowing proniosomal powder of acemetacin. These tablets showed improved pharmacokinetic characteristics, such as AUC, T_max_, half-life, and relative bioavailability, when tested in animal models (rabbits), and there was substantial increase in hardness, disintegration time, dissolution properties, and less friability of proniosomal tablet as compared to both proniosomal powders and conventional oral tablets of acemetacin (Shehata et al., 2015). Proniosomes also have a role in combination therapy with improved patient compliance as depicted from a study conducted to encapsulate both doxycycline hydrochloride (DH) and metronidazole (MT) in proniosomal carrier system by using different types of spans, cholesterol, and maltodextrin as carrier. Both drugs were successfully dispersed in proniosomal structure without any chemical interaction between them and other components. The proniosomal powder had free flowing properties and was stable at 2–8 °C for three months. Besides, these dry free flowing powder ensured easy handling and accurate dosing, avoided the hydrolysis of drugs and physical stability problems related to niosomes (Gad et al., 2014).

Proniosomes have potential to fabricate on large scale production because of their convenient preparation technology and capability to modify the drug delivery (Yuksel et al., 2016). Although, proniosomes have pronounced role in oral drug delivery but for oral delivery proniosomal powder need to be modified in the proper dosage form, such as tablets, capsules, beads, etc. To make oral proniosomal tablets palatable coating may require, therefore, cost of production may increase.

#### Parenteral delivery

Parenteral administration is a general approach usually utilized for those pharmaceutical ingredients having poor bioavailability and narrow therapeutic index. Parenteral route has enormous advantages in emergency clinical situations, such as easily accessible, rapid onset of action, favorable in conditions when oral route is not convenient including difficulty in swallowing, delayed gastric emptying and intestinal motility, vomiting, and unconsciousness. Although parenteral route of administration is the most effectual route but there is an instant decline in systemic concentrations and requires frequent administration to maintain constant therapeutic concentration of a drug that eventually results in poor patient compliance. These limitations can be avoided by utilizing biodegradable polymeric nanosystems by maintaining controlled and slow release of drug for longer durations, consequently reduces the dosing frequency and toxic effects and improve the quality of treatment (Komal et al., 2013; Din et al., 2017; Ud Din et al., 2017).

Major advancements have acquired in the field of vesicular drug delivery systems that have a potential to maintain sustained drug release via parenteral administration and to circumvent the issues related to conventional parenteral drug delivery systems, particularly associated with drugs having narrow therapeutic index and poor bioavailability (Marianecci et al., 2014). Among vesicular drug carriers, proniosomes have capability to be used to administer drugs via parenteral route as these can be stored, transported, distributed, appropriate to sterilize, and separated into unit doses for parenteral delivery (Verma et al., 2016). A parenteral proniosome formulation could maintain constant plasma concentration, improve efficacy and reduce toxicity by circumventing the frequent administrations or constant intravenous infusions which is a pronounced obstacle in improving patient compliance.

The anti-inflammatory effects of flurbiprofen were improved by fabricating its free-flowing proniosome formulation and by injecting intravenously after reconstitution. Furthermore, this formulation sustained the systemic concentration of drug by reducing the fluctuations in plasma levels which was resulted by frequent administrations and ultimately enhances its anti-inflammatory and analgesic effects. Therefore, this study proves that proniosomes are stable, affordable, propitious, and favorable alternative to other vesicular and colloidal drug delivery systems for parenteral administration as depicted from *in vivo* pharmacokinetic and pharmacodynamic effects of a rat paw edema model (Verma et al., 2016).

For parenteral delivery of nanoparticles sterilization is the main consideration. Different techniques are used for their sterilization but all have their own limitations. Freeze drying by using cryoprotectants and gamma-irradiation sterilization techniques may alter the particle size, drug release rates and difficulty in reconstitution by simple agitation (Bozdag et al., 2005). Whereas, proniosomal dry powders are free from all of these drawbacks. They can easily be sterilized; do not produce changes in the particle size and drug release rates and can be reconstituted with simple agitation.

#### Dermal and transdermal delivery

Human skin is very sensitive part of the body that covers most part of the body. The main function of skin is to maintain the hydration of body (Fukushima et al., 2011). The skin acts as a selective penetration barrier that resists the entry of certain molecules from its surface. The predominant part of the skin is the stratum corneum which is very essential in percutaneous absorption. It is a rate-limiting barrier in percutaneous absorption and shows pronounced resistance to penetration (Pathan & Setty, 2009). Drug carriers are used for targeting and delivering the drug to the specific site of action. It is dependent on the type of drug carrier that either deep skin penetration or accumulation in the stratum corneum and follicular appendages takes place (Bolzinger et al., 2011; Mak et al., 2011). Dermal drug delivery is advantageous that high concentrations confined at the site of action, diminished systemic absorption, and ultimately reducing side effects. Moreover, transdermal route has various advantages, such as: noninvasive technique, bypass first pass hepatic metabolism that eventually increases the drug bioavailability, overcomes the gastrointestinal degradation, maintains steady state plasma concentration, self-administration, and improve patient compliance (Wohlrab et al., 2011; Gupta & Babu, 2013). Besides, transdermal route shows some disadvantages, such as low permeation of some drugs through skin due to stratum corneum which is a main barrier to permeation. Vesicular drug delivery system is proved to be an alternative to circumvent the barriers of skin without other physical or chemical ways (Rajera et al., 2011).

**Figure 3.**
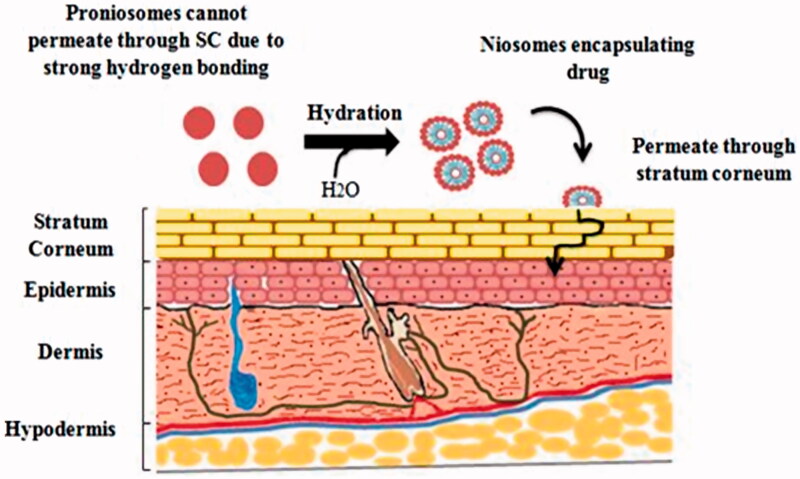
Drug delivery and penetration of proniosomes through stratum corneum.

From the last decade, excessive investigations have undergone on applications of vesicular delivery systems as carriers for dermal and transdermal drug delivery through skin and it found to be beneficial (Katare et al., 2010; Marianecci et al., 2013). Different types of vesicular drug delivery systems have been developed, such as bilosomes, pharmacosomes, emulsomes, transfersomes, liposomes, and niosomes (Kamboj et al., 2013). Proniosomes provide a flexible vesicular drug delivery concept having potential in transdermal drug delivery of drugs. Additionally, these vesicles as dermal drug delivery systems have potential to increase effectiveness and reduce toxic effects of drugs through topical application (Muzzalupo, 2016). Proniosomes are novel drug delivery system have propensity to attach to the stratum corneum, converted to niosomes after hydration and permeate in skin through stratum corneum that results in increase skin permeation (Figure 3) (Ramesh et al., 2013). These vesicular carriers acts as a drug reservoir and by the adjustment of its composition or surface drug release rate can be controlled (Kamel et al., 2013).

Several studies have been reported that depict the potential of proniosomes in dermal and transdermal delivery. The proniosomal system for transdermal delivery of simvastatin was designed to improve its oral bioavailability and *in vivo* absorption as it has bioavailability less than 5% due to substantial first pass effect and poor absorption. Proniosomal carrier system not only improved the oral bioavailability of simvastatin but also its absorption from the skin was increased that eventually results in enhanced hypocholesterolemic effects of simvastatin when compared with oral simvastatin dispersion (Shaker et al., 2013). Besides, proniosomal gel system has power to encapsulate poorly water soluble drugs, such as flurbiprofen by utilizing different nonionic surfactants and cholesterol. The effect of two factors such as nonionic surfactant fatty acid chain length and the amount of cholesterol were assessed on drug permeation and entrapment efficiency of flurbiprofen containing proniosomes. The results indicated that maximum fatty acid side chain length of nonionic surfactant and minimum cholesterol content was suitable for optimized formulation. Therefore, proniosomes provide a system with high drug entrapment and skin permeation for transdermal delivery of flurbiprofen (Zidan & Mokhtar, 2011). Mehta et al. (2016) designed topical boswellic acid (BA) loaded proniosomal gel carrier system to augment its bioavailability, absorption, and release kinetics for the management of inflammatory disorders. Boswellic acid is a leukotriene inhibitor and showed remarkable anti-inflammatory effects as compared to standard Voveran gel. Furthermore, transdermal delivery is enhanced due to small particle size and significant encapsulation of drugs within vesicles (Mehta et al., 2016). Similarly, the topical drug delivery system of Tretinoin loaded proniosomes were designed by Rahman et al. (2015) to improve the effectiveness of tretinoin by minimizing its side effects for acne treatment. Optimized formulation demonstrated less irritation to skin and higher efficacy as compared to marketed product in human models that could be due to the increased penetration of tretinoin through stratum corneum. Additionally the solubility issues, stability of tretinoin and compliance to the treatment were improved (Rahman et al., 2015).

**Figure 4.**
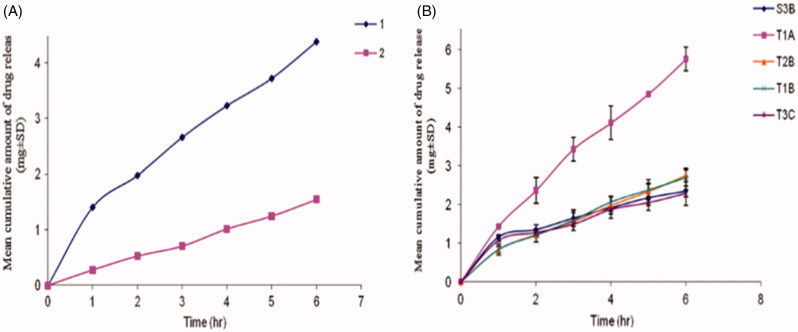
Release profile of tenoxicam from proniosomes (A) Release profile of TX from certain proniosome formulations 1 (T2B) using phosphate buffer (pH7.4) as aqueous phase; 2 (S3B) using 0.1% glycerol as aqueous phase (B) Release profile of TX from proniosomes prepared with distilled water (Will be added with permission).

Ammar et al. (2011) fabricated a proniosomal gel for transdermal delivery of tenoxicam that significantly enhanced its anti-inflammatory and analgesic effects as compared to the oral marketed tablets of tenoxicam. The release of tenoxicam was investigated for the stable formulations and the results revealed that the tenoxicam followed zero order release. The lecithin free formulation T1A hydrated by distilled water showed highest release rate and release efficiency whereas T2B hydrated by phosphate buffer (pH 7.4) showed lowest release. S3B formulation hydrated by 0.1% glycerol showed lowest release values as compared to T2B (phosphate buffer pH 7.4; Figure 4(A)). T1A was optimized and the most stable formulation showed highest release efficiency containing Tween 20, cholesterol, and distilled water as aqueous medium Figure 4(B). It was due to the fact that Tween 20 has hydrophilic nature and acts as a solubilizing agent for the drug, hence increase the drug release (Ammar et al., 2011).

El-Laithy et al. (2011) reported efficient transdermal delivery of vinpocetine via proniosomal carriers containing sugar esters as nonionic surfactants. These proniosomes are considered as convenient carrier system for delivering vinpocetine by the hydration of proniosomes into niosomes in skin. When compared to oral tablets transdermal proniosomal carriers were found to be superior as these had the potential to control the drug release and maintain minimum effective concentration up to 48 h and enhanced absorption that leads to improved therapeutic effectiveness (El-Laithy et al., 2011). The *in vivo* efficacy of mefenamic acid has been improved with transdermal drug delivery system of proniosomes. The results revealed that proniosomal gel loaded with mefenamic acid without incorporation of penetration enhancer causes considerable inhibition of rat paw edema as compared with the same control gel (Wen et al., 2014). Similarly, the proniosomal transdermal drug delivery system for lornoxicam was successfully formulated by coacervation phase separation method and evaluated for stability and acceptability for skin applications (Madan et al., 2016). Another study manifested that proniosomes are flexible drug delivery carrier system having potential to deliver drugs effectively through transdermal route. Anti-hypersensitive drug lacidipine loaded transdermal proniosomes were produced by using cremophor RH as nonionic surfactant (Soliman et al., 2016). Rajabalaya et al. (2016) successfully encapsulated oxybutynin chloride in proniosomes for its transdermal delivery in overactive bladder therapy. It was obvious from the results that proniosomes provide high drug permeation and entrapment efficiency; improve therapeutic efficacy and rapid salivary secretion recovery (Rajabalaya et al., 2016). Similarly, transdermal proniosomes were designed incorporating tolterodine tartrate (TT) for the management of overactive bladder (OAB). The side effects of transdermal delivery proniosomal gel were compared with oral formulation of tolterodine tartrate. The results demonstrated that TT transdermal proniosomal formulation has comparable efficacy to oral formulation as it produce less dry mouth effects and showed increased permeation across skin (Rajabalaya et al., 2016). An Antipsychotic drug, i.e. risperidone was also effectively delivered by proniosomal carrier system via transdermal route for the treatment of schizophrenia (Imam et al., 2015). Thus, proniosomes are favorable system for inexpensive transdermal delivery of variety of drugs because they have absorptive and penetration enhancing properties, riskless for human use, alternative, and preferable carrier over other conventional gels. Proniosomal gels can be incorporated in patches, films, and gel carriers to impart them bio adhesive properties that ultimately further enhances their contact time on skin, which if not controlled may results in local tissue damage.

#### Oral mucosal delivery

Oral mucosa has many properties that make it an acceptable site for delivery of various drugs but it offers certain problems for researchers exploring novel drug delivery techniques to overcome. Several mucosal surfaces have been examined as delivery routes including buccal, nasal, rectal, ocular, and vaginal (Hearnden et al., 2012). Oral mucosa is readily available and more permeable route than the skin, self-administrable, has extensive blood supply, less responsive to irritants, availability of more hydrated environment for drug solubility, and rapid systemic drug delivery is possible (Sankar et al., 2011).

Proniosomes have been extensively investigated for mucosal delivery of drugs through oral cavity and have a potential for systemic delivery of drugs across the mucosal membrane. Besides this, they suffer through poor retention at application site in the oral cavity due to instant wash over by saliva. Therefore, the contact time with underlying mucosal surfaces can be enhanced by incorporating proniosomal gel into mucoadhesive carbopol base gel to achieve effective therapeutic concentration for longer period of time (Abdelbary & Aburahma, 2015).

Abdelbary & Aburahma (2015) successfully prepared oro-dental and anti-inflammatory mucoadhesive proniosomal gel loaded with lornoxicam to deliver drug directly at site of action in the oral cavity having dental pain and inflammation. This provesicular approach is valuable in augmenting the therapeutic efficacy of lornoxicam for dental delivery that results in better patient compliance and reduced gastrointestinal adverse effects. The results of the study showed that the oral mucoadhesive proniosomal gels can be considered as a favorable approach for transmucosal delivery of lornoxicam into the oral cavity (Abdelbary & Aburahma, 2015). In another study, proniosomes were successfully prepared for buccal delivery of benzocaine as an effective, long acting vesicular formulation for improving local anesthesia in the buccal cavity. The *in vitro* release behavior and *ex vivo* permeation studies of proniosomal benzocaine gel through buccal mucosa confirmed a controlled release of benzocaine for management of mucosal pain for longer duration of time (El-Alim et al., 2014). To provide the mucoadhesive properties proniosomal gels are incorporated in carbopol base gel as discussed in the above studies. However, the mucoadhesion time can be improved further by incorporating them in other mucoadhesive polymers, such as thiolated polymer conjugates. The adhesion time and the total work of adhesion (TWA) of thiolated polymers are considerably high than the carbopol polymers (Grabovac et al., 2005).

#### Ocular delivery

Ocular drug delivery system is one of the most peculiar and challenging route for formulation scientists. An ocular formulation should release the drug by bypassing the protective barriers of the eye without damaging the tissues (Ali et al., 2016). Ocular drug delivery systems generally have low bioavailability, limited absorption in the intraocular area due to blinking reflex, less eye capacity, nasolacrimal drainage, and the corneal and conjunctival epithelial barriers which ultimately reduces the retention time of drug molecules in the eyes (Kaur et al., 2012). Thus, frequent instillations of eye drops are usually required in clinical systems to attain anticipated therapeutic effectiveness which leads to patient noncompliance, tissue damage, and other toxic effects integrated with nasolacrimal absorption (Luo et al., 2011).

To overcome the drawbacks of conventional eye preparations, various ocular drug delivery systems have been explored recently, such as *in situ* gels (Gupta et al., 2015), liposomes (Dai et al., 2013), nanospheric suspensions (Fei et al., 2008), *in situ* nanosuspensions (Luschmann et al., 2013), nanoparticles (Nagarwal et al., 2012), nanoemulsions (Garg et al., 2013), and niosomes (Abdelkader et al., 2012). Among all of these, proniosomes have strong potential in topical ocular drug delivery. Proniosomes can form niosomes after hydration in ocular cavity and both nonionic surfactants and phospholipids in proniosomes act as penetration enhancers (Ammar et al., 2011). Furthermore, proniosomal gels as ocular formulation can increase the residence time of the drugs in corneal cavity, provide prolonged and sustained action, prevent enzymatic metabolism in tears, and in corneal epithelial surface thus, improve ocular bioavailability (Dhangar et al., 2014; Li et al., 2014).

Li et al. (2014) prepared a proniosomal system for ophthalmic delivery of tacrolimus that resulted in formation of niosomes after hydration in ocular cavity. The *in vitro* permeation of tacrolimus loaded proniosomes was performed on freshly excised rabbit cornea and it showed enhanced permeation due to increased retention of tacrolimus in cornea as compared to conventional ointments. Tacrolimus loaded proniosomes retard the corneal allograft rejection and sustained the survival time of corneal allografts considerably than Cyclosporine eye drops in Sprague–Dawley rat corneal xeno transplantation model (Li et al., 2014). In another study, proniosomal gel system was developed by encapsulating Lomefloxacin HCl to enhance its ocular bioavailability for the treatment of bacterial conjunctivitis. The developed optimized system prolongs the retention time of the drug in the cornea, increase local effect, enhance penetration, and control the release of drug that eventually leads to increased antibacterial therapeutic effects. Additionally, proniosomal gel was found to be safe, stable, and superior to commercially available eye drops of Lomefloxacin HCl. Hence, it is a favorable approach for management of bacterial conjunctivitis (Khalil et al., 2016).

#### Vaginal delivery

The vaginal route of drug delivery is usually employed for the treatment of microbial infections, such as vulvovaginal candidiasis and bacterial vaginosis (Hainer & Gibson, 2011). This route is extensively used for the delivery of various molecules, such as antimicrobials, antimycotics, sexual hormones, and peptides as it is highly vascularized and has high permeability. Moreover, it bypasses the hepatic first-pass metabolism and overcome the gastrointestinal absorption that is why it is an outstanding route for mucosal drug delivery for both local and systemic purposes (Ensign et al., 2014). In spite of these advantages, vaginal route has some peculiarities that cause variations in the bioavailability of drugs. Various physiological and non-physiological factors, such as menstrual cycle or menopause, sexual intercourse, and personal hygiene can provoke changes in vaginal conditions (Vanić and Škalko-Basnet, 2013; Yang et al., 2013). Additionally, the natural clearance process by vaginal secretions, i.e. mucus also restricts the actions of vaginal drug delivery systems (das Neves et al., 2011).

In vaginal drug delivery tablets, solutions, foams, vaginal suppositories and inserts have been frequently used but semisolid dosage forms especially gels are preferred considerably (Neves et al., 2014). However, conventional vaginal systems have certain restrictions, such as leakage, short residence time and disarray that can be controlled by novel formulations showing adequate retention, sufficient release, and diffusion characteristics. Certain micro and nano novel delivery systems have been offered including hydrogels (Khade et al., 2014), vaginal sponges (Furst et al., 2015), nanoparticles (Meng et al., 2011; Date et al., 2012), nanocapsules (Santos et al., 2013), solid lipid NPs (Alukda et al., 2011), liposomes (Vanić et al., 2014), and niosomes (Ning et al., 2005).

The vaginal drug delivery systems that have been mostly evaluated are mucoadhesive systems which retains the drug in the vaginal mucosa by close contact of formulation with vaginal mucosa and thus maintain the adequate concentration of the drug in the targeted area (de Araújo Pereira & Bruschi, 2012). Proniosomal gel systems are convenient carrier system for vaginal drug delivery having good mucoadhesive properties and provide constant release of the drug. Abdou & Ahmed (2016) designed a mucoadhesive system for vaginitis encapsulating antifungal drug, i.e. terconazole. The proniosomal system hydrated to form niosomes and provides an effective treatment against vaginitis by sustained release of drug and enhanced retention at the vaginal mucosa. Optimized proniosomal formulation was incorporated in carbopol gel to increase its mucoadhesive properties and microbiological evaluation showed that proniosomes are efficient vesicular system for mucosal drug delivery (Abdou & Ahmed, 2016). The matter of concerns regarding the vaginal proniosomal delivery is the residence time of the mucoadhesive systems. It should be adequate otherwise; they may irritate the mucosal surface and may leads to severe local tissue damage.

#### Pulmonary delivery

Lungs gained considerable attention for treating various respiratory diseases as it is the main organ responsible for respiratory disorders (Misra et al., 2011). Inhalation route provides a targeted drug delivery to the lungs for various respiratory diseases. It assures local delivery of the drugs to the lungs by reducing systemic exposure as with oral and parenteral route. Thus, low doses are required to produce the therapeutic effect and ultimately reduce systemic adverse effects especially for drugs having narrow therapeutic index. Lungs have increased drug absorption and efficacy due to large surface area (>100 m^2^) and is covered with epithelial layer (Nahar et al., 2013). The lungs are the effective way of entry to the systemic circulation by the presence of large surface area of the alveoli, extensive blood flow in the lungs, narrow diffusion path between the blood and alveoli and avoidance of first pass hepatic effect. Besides, this route becomes more convenient for the patients by the use of portable inhalers (Zhou et al., 2014). Nevertheless, the rapid clearance of the inhaled drugs in the lungs leads to less therapeutic effectiveness (Todoroff & Vanbever, 2011).

With advancements in nanotechnology, the treatment of various lung diseases by inhalation route has been improved. Various nano drug delivery systems are employed to target the specific tissues and cells in order to achieve enhanced effects and reduce side effects of the drug in the lungs and other organs (Kuzmov & Minko, 2015). Nebulizable delivery systems of proniosomes were successfully designed by incorporating beclomethasone dipropionate (BDP) as model drug. Two systems were developed that are Aeroneb pro and Omron Micro air vibrating-mesh nebulizer and Pari LC Sprint air-jet nebulizer. The aerosol properties of the niosomes formed after hydration of proniosomes were analyzed. The results revealed that the entrapment of BDP in proniosomes was higher as compared to the conventional niosomes. Moreover, high drug output and fine particle fraction (FPF) was achieved by aerosols produced from both nebulizable systems. Therefore, proniosomal system manifested as feasible methodology to deliver BDP-niosomes through vibrating-mesh and air jet nebulizers (Elhissi et al., 2013).

In another study, Abd-Elbary et al. (2008) successfully developed a controlled release nebulizable delivery system for cromolyn sodium loaded in proniosomes by using nonionic biocompatible and biodegradable surfactant, i.e. sucrose stearate. These observations evident that proniosomes are feasible, stable promptly hydrated, and dry free flowing carrier system for nebulization therapy of cromolyn sodium (Abd-Elbary et al., 2008).

#### Intranasal delivery

Intranasal delivery is a promising noninvasive technique to deliver the drugs directly to the CNS by avoiding the blood brain barrier (BBB). The highly permeable nasal epithelium, porous endothelial membrane, large surface area, and high blood flow permit prompt absorption of drug in the nasal mucosa. Various therapeutic drugs can be delivered to the CNS by intranasal route (Chopra et al., 2011). Intranasal route is superior to other conventional drug delivery methods and has prominent advantages in treating different CNS diseases. Intranasal route is noninvasive system that can reduce the pain of injection and it is convenient route for patients as it can be conducted by a patient itself. Moreover, large molecules can be delivered to the brain effectively by crossing the BBB; small doses are required to produce the appropriate concentration in the CNS and it can reduce the toxic effects of the drugs on peripheral system that ultimately reduces the cost of therapy (Bhatt et al., 2015). Intranasal delivery is also an alternative to other invasive delivery routes such as cerebro ventricular or intra-parenchymal and deliver drugs directly from nasal mucosa to the CNS effectively by employing olfactory and trigeminal pathways to reduce systemic exposure (Bahadur & Pathak, 2012). However, nasal delivery has some demerits such as rapid removal of drugs due to mucociliary clearance system, mucosal damage on repeated use of this route, nasal irritation, nasal congestion, and partial degradation by nasal peptidase enzyme system (Ali et al., 2010).

Currently, the most advanced and favorable noninvasive approach for brain delivery is the development of nanocarriers having size range between 1 and 100 nm which can easily cross the brain endothelium. Furthermore, nanocarriers have the ability to keep drugs away from biological environment and avoid enzymatic degradation and thereby enhance the cellular uptake and drug bioavailability. Thus, it is an important therapeutic approach for treating various neurological disorders and minimizing the adverse effects of several therapeutic drugs (Gurrapu et al., 2012; Santos et al., 2012). Vesicular drug delivery systems (liposomes and niosomes) are promising drug delivery carriers that have the capability to deliver both small and large therapeutic agents effectively through intranasal route to the CNS by circumventing the restrictions of nasal delivery including mucociliary clearance, enzymatic degradation, and increase drug bioavailability (Alsarra et al., 2010). The utilization of these drug carriers is mainly due to their versatile nature, the chemical properties associated with surfactants and phospholipids employed for their preparation (Agrati et al., 2011). It can be concluded that, proniosomes have the potential to deliver drugs through intranasal route, as these have penetration and absorption enhancing properties. The delivery of proniosomes through intranasal route has not been reported yet.

### Toxicities associated with proniosomes

From the above discussion, it is concluded that the safety profile of proniosomes is quite good because of the components that are used to fabricate them are safe and biocompatible. Although surfactants are supposed to illustrate toxicities when they are used to develop drug delivery systems, however, practically adequate data is not available about the toxicities associated with proniosomes (Yadav et al., 2011). Proniosomes does not produce any signs of toxicity when administered through oral and parenteral routes. Lornoxicam (NSAID) toxicity is dose related when applied at the desired site of action. Oro-dental mucoadhesive proniosomal gel is a favorable approach that improves the safety of lornoxicam in dental application (Abdelbary & Aburahma, 2015). The ocular irritancy test of lomefloxacin proniosomes showed that they have high ocular tolerability and does not produce redness and inflammation in the eye (Khalil et al., 2016). Another study showed that nonionic surfactants may damage corneal and conjunctival epithelium and produce ocular irritation, redness, and discharge (Maurer et al., 1997; Maurer et al., 1998) whereas (Maiti et al., 2011) reported that common excipients used in NSVs, such as span 60 and cholesterol are safe and does not produce any ocular toxic sign (Maiti et al., 2011). Thus, non-ionic surfactant vesicles including proniosomes are safe and do not produce any ciliotoxicity and cytotoxicity as depicted from a study conducted to assess the safety of NSVs for topical delivery. Ciliotoxicity model is used for the safety assessment of intranasal formulations whereas; cytotoxicity model is used for skin delivery. Both models revealed that NSVs are safe in all respects because of their physicochemical properties (Hofland et al., 1992).

### Proniosomes versus niosomes

From decades, vesicular systems have been used as drug delivery carriers for multiple purposes, such as targeted drug delivery, increased drug transport through different biological barriers, and controlled drug release. Vesicular drug delivery systems include liposomes, niosomes, transfersomes, ethosomes, and others. These systems are similar to the conventional liposomes but vary in structure and function (El Maghraby et al., 2008). Liposomes and niosomes have various applications in drug delivery but they experienced a lot of demerits, such as instability due to phospholipids degradation and aggregation, fusion, and leakage of drug. Proniosomes have the potential to overcome these drawbacks as they can be sterilized, stored at room temperature and can be hydrated instantly to form niosomal dispersion before administration.

Proniosome powders have prominent advantages and are also preferred over lyophilized powders as the drug properties are altered by lyophilization. Several published studies reported the superiority of proniosomes over niosomes. A comparative study conducted on stability studies of niosomes and proniosomes demonstrated that proniosomes can be effectively stored at room temperature and the drug leakage from the proniosome vesicles was reduced which is the main concern with noisome when stored at room temperature (Ruckmani et al., 2010). Similarly, the stability of proniosomes can be manifested by the assessment of proniosomal formulations for three months containing tenoxicam. The results of stability studies revealed higher entrapment efficiency and retention and no considerable change was observed in mean particle size when compared to freshly prepared sucrose stearate noisome over a period of 90 d (Ammar et al., 2011). Furthermore, a proniosomal gel emerged as a propitious system to deliver estradiol effectively through transdermal route with enhanced skin permeability as compared to niosomes. The enhanced permeation of estradiol across skin is attributed to the fact that proniosomes contain nonionic surfactants and lecithin which has penetration enhancing properties (Fang et al., 2001).

## Conclusion

With recent advances in drug delivery systems proniosomes have gained pronounced recognition in the field of drug delivery. Proniosomes are a versatile drug delivery system having a number of advantages over other drug delivery carriers, such as liposomes and niosomes. Proniosomes are the dry formulations that promptly form niosomes upon hydration. Niosomes formed from proniosomal technology are superior over conventional niosomes in terms of stability and the hydration of dry proniosomes is extremely simple than the extensive shaking processes involved in standard film hydration method. They have the potential to attenuate the solubility and permeability problems of class II and IV drugs and have the capability to incorporate both hydrophilic and hydrophobic drugs. Furthermore, they have the scalable properties and unit dosage forms can be developed from dry powder proniosomes including tablets, beads, and capsules. They provide further benefit of transportation, distribution and storage, and dose.

Extensive research reported on proniosomes evident their effectiveness in drug delivery and targeting. Proniosomes are suitable carrier system for the delivery of wide variety of drugs through different routes, such as oral, parenteral, dermal, transdermal, ocular, vaginal, mucosal pulmonary, and nasal effectively. Proniosomes are extensively employed in oral and transdermal delivery of wide variety of drugs. In oral delivery, they predominantly used to improve the bioavailability and absorption from the gastrointestinal tract. Moreover, they have a promising role in transdermal delivery because of their penetration enhancing properties, non-toxicity, and drug release modulation properties. While in mucosal drug delivery, they gained appreciable attention and provide new aspects in the field of pharmaceutical research. Furthermore, proniosomes have wide applications in drug delivery and targeting and has opened the door of research for other active pharmaceutical agents including anticancer drugs, vaccines, and genes.

## Future perspectives

From last few decades, the concept of proniosome derived-niosomes have been introduced new aspects in pharmaceutical research and the most accepted by the research scientists in targeting the specific organs or tissues for better therapeutic effects. Proniosomes is the modern concept that opens the door of research in pharmaceutical field. Different new carrier materials can be analyzed for future use in the production of proniosomes that are biocompatible and appropriate for proniosomes. Moreover, proniosomes are becoming promising drug delivery carrier among vesicular systems but there is a need to explore them in the field of nutraceuticals, herbal compounds, and cosmetics. They are also suitable for the delivery of peptides as peptides undergo enzymatic degradation when administered through oral route due to the presence of enzymes and acidic condition. Higher stability of peptides could be achieved by proniosomal technology. Additionally, they are convenient for the delivery of vaccines and antigens and could function better in presenting the antigens to antigen identification cells.

Further, the drugs having pronounced adverse effects can be effectively delivered by proniosomal carriers to improve their therapeutic effectiveness by minimizing side effects. Proniosomes could also be used to deliver hemoglobin within the blood as they are permeable to oxygen and, therefore, can be effectively used as a carrier for the treatment of anemia. Hence, an extensive research is required to explore them in industrial set up by developing pilot plant scale up studies. Yet, there are many challenges that need to be assessed in industrial system and prove their suitability for the delivery of wide range of drugs and natural products.

## References

[CIT0001] Abd-Elbary A, El-Laithy H, Tadros M. (2008). Sucrose stearate-based proniosome-derived niosomes for the nebulisable delivery of cromolyn sodium. Int J Pharm 357:189–98. 18339494 10.1016/j.ijpharm.2008.01.056

[CIT0002] Abdelbary GA, Aburahma MH. (2015). Oro-dental mucoadhesive proniosomal gel formulation loaded with lornoxicam for management of dental pain. J Liposome Res 25:107–21. 25058447 10.3109/08982104.2014.941861

[CIT0003] Abdelkader H, Wu Z, Al-Kassas R, Alany RG. (2012). Niosomes and discomes for ocular delivery of naltrexone hydrochloride: morphological, rheological, spreading properties and photo-protective effects. Int J Pharm 433:142–8. 22595640 10.1016/j.ijpharm.2012.05.011

[CIT0004] Abdou EM, Ahmed NM. (2016). Terconazole proniosomal gels: effect of different formulation factors, physicochemical and microbiological evaluation. J Pharm Drug Deliv Res 5:1.

[CIT0005] Aburahma MH, Abdelbary GA. (2012). Novel diphenyl dimethyl bicarboxylate provesicular powders with enhanced hepatocurative activity: preparation, optimization, *in vitro/in vivo* evaluation. Int J Pharm 422:139–50. 22079716 10.1016/j.ijpharm.2011.10.043

[CIT0006] Agrati C, Marianecci C, Sennato S, et al. (2011). Multicompartment vectors as novel drug delivery systems: selective activation of Tγδ lymphocytes after zoledronic acid delivery. Nanomed Nanotechnol Biol Med 7:153–61. 10.1016/j.nano.2010.10.003 21034859

[CIT0007] Ahmad MZ, Alkahtani SA, Akhter S, et al. (2016). Progress in nanotechnology-based drug carrier in designing of curcumin nanomedicines for cancer therapy: current state-of-the-art. J Drug Targeting 24:273–93. 10.3109/1061186X.2015.1055570 26066739

[CIT0008] Ahmad MZ, Mohammed AA, Mokhtar Ibrahim M. (2017). Technology overview and drug delivery application of proniosome. Pharm Dev Technol 22:302–11. 26794727 10.3109/10837450.2015.1135344

[CIT0009] Akhilesh D, Faishal G, Kamath J. (2012). Comparative study of carriers used in proniosomes. Int J Pharm Chem Sci 3:6–12.

[CIT0010] Akhilesh D, Hazel G, Kamath J. (2011). Proniosomes–A propitious provesicular drug carrier. Int J Pharm Pharm Sci Res 1:98–103.

[CIT0011] Ali J, Ali M, Baboota S, et al. (2010). Potential of nanoparticulate drug delivery systems by intranasal administration. Curr Pharm Design 16:1644–53. 10.2174/138161210791164108 20210751

[CIT0014] Ali J, Fazil M, Qumbar M, et al. (2016). Colloidal drug delivery system: amplify the ocular delivery. Drug Deliv 23:700–16. 10.3109/10717544.2014.923065 24892625

[CIT0012] Alsarra IA, Hamed AY, Alanazi FK, El Maghraby GM. (2010). Vesicular systems for intranasal drug delivery. In: Drug delivery to the central nervous system. New York: Springer, 175–203.

[CIT0013] Alukda D, Sturgis T, Youan BBC. (2011). Formulation of tenofovir‐loaded functionalized solid lipid nanoparticles intended for HIV prevention. J Pharm Sci 100:3345–56. 21437910 10.1002/jps.22529 PMC3375324

[CIT0015] Ammar H, Ghorab M, EL-Nahhas S, Higazy I. (2011). Proniosomes as a carrier system for transdermal delivery of tenoxicam. Int J Pharm 405:142–52. 21129461 10.1016/j.ijpharm.2010.11.003

[CIT0016] Bahadur S, Pathak K. (2012). Physicochemical and physiological considerations for efficient nose-to-brain targeting. Expert Opin Drug Deliv 9:19–31. 22171740 10.1517/17425247.2012.636801

[CIT0017] Banerjee A, Onyuksel H. (2012). Peptide delivery using phospholipid micelles. Wires Nanomed Nanobiotechnol 4:562–74. 10.1002/wnan.1185 22847908

[CIT0018] Bhatt R, Singh D, Prakash A, Mishra N. (2015). Development, characterization and nasal delivery of rosmarinic acid-loaded solid lipid nanoparticles for the effective management of Huntington’s disease. Drug Deliv 22:931–9. 24512295 10.3109/10717544.2014.880860

[CIT0019] Bochot A, Fattal E. (2012). Liposomes for intravitreal drug delivery: a state of the art. J Control Release 161:628–34. 22289436 10.1016/j.jconrel.2012.01.019

[CIT0020] Bolzinger MA, Briançon S, Chevalier Y. (2011). Nanoparticles through the skin: managing conflicting results of inorganic and organic particles in cosmetics and pharmaceutics. Wiley Interdiscip Rev 3:463–78. 10.1002/wnan.146 21618448

[CIT0021] Bozdag S, Dillen K, Vandervoort J, Ludwig A. (2005). The effect of freeze‐drying with different cryoprotectants and gamma‐irradiation sterilization on the characteristics of ciprofloxacin HCl‐loaded poly (D, L‐lactide‐glycolide) nanoparticles. J Pharm Pharmacol 57:699–707. 15969924 10.1211/0022357056145

[CIT0022] Caddeo C, Pons R, Carbone C, et al. (2017). Physico-chemical characterization of succinyl chitosan-stabilized liposomes for the oral co-delivery of quercetin and resveratrol. Carbohydr Polym 157:1853–61. 27987905 10.1016/j.carbpol.2016.11.072

[CIT0023] Chaw CS, Ah Kim KY. (2013). Effect of formulation compositions on niosomal preparations. Pharm Dev Technol 18:667–72. 22468904 10.3109/10837450.2012.672988

[CIT0024] Chen H, Chi X, Li B, et al. (2014a). Drug loaded multilayered gold nanorods for combined photothermal and chemotherapy. Biomater Sci 2:996–1006. 32481973 10.1039/c3bm60323g

[CIT0025] Chen H, Li B, Qiu J, et al. (2013). Thermal responsive micelles for dual tumor-targeting imaging and therapy. Nanoscale 5:12409–24. 24165905 10.1039/c3nr04529c

[CIT0026] Chen H, Li B, Zhang M, et al. (2014b). Characterization of tumor-targeting Ag2S quantum dots for cancer imaging and therapy *in vivo*. Nanoscale 6:12580–90. 25184523 10.1039/c4nr03613a

[CIT0027] Chopra K, Misra S, Kuhad A. (2011). Neurobiological aspects of Alzheimer's disease. Expert Opin Ther Targets 15:535–55. 21314231 10.1517/14728222.2011.557363

[CIT0028] Dai Y, Zhou R, Liu L, et al. (2013). Liposomes containing bile salts as novel ocular delivery systems for tacrolimus (FK506): *in vitro* characterization and improved corneal permeation. Int J Nanomedicine 8:1921–33. 23690687 10.2147/IJN.S44487 PMC3656938

[CIT0029] Das Neves J, Amiji M, Sarmento B. (2011). Mucoadhesive nanosystems for vaginal microbicide development: friend or foe? Wiley Interdiscip Rev Nanomed Nanobiotechnol 3:389–99. 21506290 10.1002/wnan.144

[CIT0030] Date AA, Shibata A, Goede M, et al. (2012). Development and evaluation of a thermosensitive vaginal gel containing raltegravir + efavirenz loaded nanoparticles for HIV prophylaxis. Antiviral Res 96:430–6. 23041201 10.1016/j.antiviral.2012.09.015 PMC3513487

[CIT0031] De Araújo Pereira RR, Bruschi ML. (2012). Vaginal mucoadhesive drug delivery systems. Drug Dev Ind Pharm 38:643–52. 21999572 10.3109/03639045.2011.623355

[CIT0032] Dhangar R, Bhowmick M, Parihar SS, et al. (2014). Design and evaluation of proniosomes as drug carrier for ocular delivery of levofloxacin. J Drug Deliv Ther 4:182–9.

[CIT0033] Din FU, Choi JY, Kim DW, et al. (2017). Irinotecan-encapsulated double-reverse thermosensitive nanocarrier system for rectal administration. Drug Deliv 24:502–10. 28181835 10.1080/10717544.2016.1272651 PMC8241086

[CIT0034] El-Alim SA, Kassem A, Basha M. (2014). Proniosomes as a novel drug carrier system for buccal delivery of benzocaine. J Drug Deliv Sci Technol 24:452–8.

[CIT0035] El-Laithy HM, Shoukry O, Mahran LG. (2011). Novel sugar esters proniosomes for transdermal delivery of vinpocetine: preclinical and clinical studies. Eur J Pharm Biopharm 77:43–55. 21056658 10.1016/j.ejpb.2010.10.011

[CIT0036] El Maghraby GM, Ahmed AA, Osman MA. (2015). Penetration enhancers in proniosomes as a new strategy for enhanced transdermal drug delivery. Saudi Pharm J 23:67–74. 25685045 10.1016/j.jsps.2014.05.001 PMC4310994

[CIT0037] El Maghraby GM, Barry BW, Williams AC. (2008). Liposomes and skin: from drug delivery to model membranes. Eur J Pharm Sci 34:203–22. 18572392 10.1016/j.ejps.2008.05.002

[CIT0038] Elhissi A, Hidayat K, Phoenix DA, et al. (2013). Air-jet and vibrating-mesh nebulization of niosomes generated using a particulate-based proniosome technology. Int J Pharm 444:193–9. 23299083 10.1016/j.ijpharm.2012.12.040

[CIT0039] Ensign LM, Cone R, Hanes J. (2014). Nanoparticle-based drug delivery to the vagina: a review. J Control Release 190:500–14. 24830303 10.1016/j.jconrel.2014.04.033 PMC4142075

[CIT0040] Fang JY, Yu SY, Wu PC, et al. (2001). *In vitro* skin permeation of estradiol from various proniosome formulations. Int J Pharm 215:91–9. 11250095 10.1016/s0378-5173(00)00669-4

[CIT0041] Fei WL, Chen JQ, Yuan J, et al. (2008). Preliminary study of the effect of FK506 nanospheric-suspension eye drops on rejection of penetrating keratoplasty. J Ocular Pharmacol Ther 24:235–44. 10.1089/jop.2007.0059 18321198

[CIT0042] Fukushima K, Ise A, Morita H, et al. (2011). Two-layered dissolving microneedles for percutaneous delivery of peptide/protein drugs in rats. Pharm Res 28:7–21. 20300802 10.1007/s11095-010-0097-7

[CIT0043] Furst T, Piette M, Lechanteur A, et al. (2015). Mucoadhesive cellulosic derivative sponges as drug delivery system for vaginal application. Eur J Pharm Biopharm 95:128–35. 25660908 10.1016/j.ejpb.2015.01.019

[CIT0044] Gad HA, Kamel AO, Sammour OA, El Dessouky HF. (2014). Vesicular powder as carrier for doxycycline hydrochloride and metronidazole combination therapy. Pharm Dev Technol 19:755–68. 23981239 10.3109/10837450.2013.829098

[CIT0045] Garg V, Jain GK, Nirmal J, Kohli K. (2013). Topical tacrolimus nanoemulsion, a promising therapeutic approach for uveitis. Med Hypotheses 81:901–4. 24018283 10.1016/j.mehy.2013.08.007

[CIT0046] Grabovac V, Guggi D, Bernkop-Schnürch A. (2005). Comparison of the mucoadhesive properties of various polymers. Adv Drug Deliv Rev 57:1713–23. 16183163 10.1016/j.addr.2005.07.006

[CIT0047] Gupta H, Aqil M, Khar R, et al. (2015). An alternative in situ gel-formulation of levofloxacin eye drops for prolong ocular retention. J Pharm Bioall Sci 7:9. 10.4103/0975-7406.149810 PMC4333635 25709330

[CIT0048] Gupta H, Babu R. (2013). Transdermal delivery: product and patent update. DDF 7:184–205. 10.2174/187221130703131128121747 24025130

[CIT0049] Gurrapu A, Jukanti R, Bobbala SR, et al. (2012). Improved oral delivery of valsartan from maltodextrin based proniosome powders. Adv Powder Technol 23:583–90.

[CIT0050] Hainer BL, Gibson MV. (2011). Vaginitis: diagnosis and treatment. Am Fam Physician 83:807–15. 21524046

[CIT0051] Hearnden V, Sankar V, Hull K, et al. (2012). New developments and opportunities in oral mucosal drug delivery for local and systemic disease. Adv Drug Deliv Rev 64:16–28. 21371513 10.1016/j.addr.2011.02.008

[CIT0052] Hofland H, Bouwstra J, Verhoef J, et al. (1992). Safety aspects of non‐ionic surfactant vesicles: a toxicity study related to the physicochemical characteristics of non‐ionic surfactants. J Pharm Pharmacol 44:287–94. 1355538 10.1111/j.2042-7158.1992.tb03608.x

[CIT0053] Hu C, Rhodes DG. (1999). Proniosomes: a novel drug carrier preparation. Int J Pharm 185:23–35. 10425362 10.1016/s0378-5173(99)00122-2

[CIT0054] Hua S, Wu SY. (2013). The use of lipid-based nanocarriers for targeted pain therapies. Front Pharmacol 4:143. 24319430 10.3389/fphar.2013.00143 PMC3836271

[CIT0055] Imam SS, Aqil M, Akhtar M, et al. (2015). Formulation by design-based proniosome for accentuated transdermal delivery of risperidone: *in vitro* characterization and in vivo pharmacokinetic study. Drug Deliv 22:1059–70. 24471715 10.3109/10717544.2013.870260

[CIT0056] Janga KY, Jukanti R, Velpula A, et al. (2012). Bioavailability enhancement of zaleplon via proliposomes: role of surface charge. Eur J Pharm Biopharm 80:347–57. 22041602 10.1016/j.ejpb.2011.10.010

[CIT0057] Kamboj S, Saini V, Maggon N, et al. (2013). Novel vesicular drug carriers for bioavailability enhancement. Int J Pharm Sci Rev Res 22:92–7.

[CIT0058] Kamel R, Basha M, Abd El-Alim SH. (2013). Development of a novel vesicular system using a binary mixture of sorbitan monostearate and polyethylene glycol fatty acid esters for rectal delivery of rutin. J Liposome Res 23:28–36. 23083098 10.3109/08982104.2012.727422

[CIT0059] Katare OP, Raza K, Singh B, Dogra S. (2010). Novel drug delivery systems in topical treatment of psoriasis: rigors and vigors. Indian J Dermatol Venereol Leprol 76:612. 21079304 10.4103/0378-6323.72451

[CIT0060] Kaur IP, Rana C, Singh M, et al. (2012). Development and evaluation of novel surfactant-based elastic vesicular system for ocular delivery of fluconazole. J Ocular Pharmacol Ther 28:484–96. 10.1089/jop.2011.0176 22694593

[CIT0061] Khade S, Behera B, Sagiri S, et al. (2014). Gelatin–PEG based metronidazole-loaded vaginal delivery systems: preparation, characterization and in vitro antimicrobial efficiency. Iran Polym J 23:171–84.

[CIT0062] Khalil RM, Abdelbary GA, Basha M, et al. (2016). Design and evaluation of proniosomes as a carrier for ocular delivery of lomefloxacin HCl. J Liposome Res 27:1–12. 27079800 10.3109/08982104.2016.1167737

[CIT0063] Khan MI, Madni A, Peltonen L. (2016). Development and *in-vitro* characterization of sorbitan monolaurate and poloxamer 184 based niosomes for oral delivery of diacerein. Eur J Pharm Sci 95:88–95. 27600819 10.1016/j.ejps.2016.09.002

[CIT0064] Komal R, Mahendra G, Shivraj P, Vinod A. (2013). Novel trends in parenteral drug delivery system: review. Int J Pharm Technol 5:2549–77.

[CIT0065] Kraft JC, Freeling JP, Wang Z, Ho RJ. (2014). Emerging research and clinical development trends of liposome and lipid nanoparticle drug delivery systems. J Pharm Sci 103:29–52. 24338748 10.1002/jps.23773 PMC4074410

[CIT0066] Kumar K, Rai A. (2012). Proniosomal formulation of curcumin having anti-inflammatory and anti-arthritic activity in different experimental animal models. Pharmazie Int J Pharm Sci 67:852–7. 23136720

[CIT0067] Kuzmov A, Minko T. (2015). Nanotechnology approaches for inhalation treatment of lung diseases. J Control Release 219:500–18. 26297206 10.1016/j.jconrel.2015.07.024

[CIT0068] Li Q, Li Z, Zeng W, et al. (2014). Proniosome-derived niosomes for tacrolimus topical ocular delivery: *in vitro* cornea permeation, ocular irritation, and in vivo anti-allograft rejection. Eur J Pharm Sci 62:115–23. 24905830 10.1016/j.ejps.2014.05.020

[CIT0069] Luo Q, Zhao J, Zhang X, Pan W. (2011). Nanostructured lipid carrier (NLC) coated with Chitosan Oligosaccharides and its potential use in ocular drug delivery system. Int J Pharm 403:185–91. 20951778 10.1016/j.ijpharm.2010.10.013

[CIT0070] Luschmann C, Tessmar J, Schoeberl S, et al. (2013). Developing an in situ nanosuspension: a novel approach towards the efficient administration of poorly soluble drugs at the anterior eye. Eur J Pharm Sci 50:385–92. 23880334 10.1016/j.ejps.2013.07.002

[CIT0071] Madan JR, Ghuge NP, Dua K. (2016). Formulation and evaluation of proniosomes containing lornoxicam. Drug Deliv Translat Res 6:511–18. 10.1007/s13346-016-0296-9 27255375

[CIT0072] Mahale N, Thakkar P, Mali R, et al. (2012). Niosomes: novel sustained release nonionic stable vesicular systems—an overview. Adv Colloid Interface Sci 183:46–54. 22947187 10.1016/j.cis.2012.08.002

[CIT0073] Maiti S, Paul S, Mondol R, et al. (2011). Nanovesicular formulation of brimonidine tartrate for the management of glaucoma: *in vitro* and *in vivo* evaluation. AAPS PharmSciTech 12:755–63. 21671199 10.1208/s12249-011-9643-9 PMC3134641

[CIT0074] Mak WC, Richter H, Patzelt A, et al. (2011). Drug delivery into the skin by degradable particles. Eur J Pharm Biopharm 79:23–7. 21457780 10.1016/j.ejpb.2011.03.021

[CIT0075] Marianecci C, Di Marzio L, Rinaldi F, et al. (2014). Niosomes from 80s to present: the state of the art. Adv Colloid Interface Sci 205:187–206. 24369107 10.1016/j.cis.2013.11.018

[CIT0076] Marianecci C, Rinaldi F, Esposito S, et al. (2013). Niosomes encapsulating ibuprofen–cyclodextrin complexes: preparation and characterization. CDT 14:1070–8. 10.2174/1389450111314090015 23531114

[CIT0077] Maurer JK, Li HF, Petroll WM, et al. (1997). Confocal microscopic characterization of initial corneal changes of surfactant-induced eye irritation in the rabbit. Toxicol Appl Pharmacol 143:291–300. 9144446 10.1006/taap.1996.8097

[CIT0078] Maurer JK, Parker RD, Carr GJ. (1998). Ocular irritation: microscopic changes occurring over time in the rat with surfactants of known irritancy. Toxicol Pathol 26:217–25. 9547859 10.1177/019262339802600205

[CIT0079] Mehta M, Dureja H, Garg M. (2016). Development and optimization of boswellic acid-loaded proniosomal gel. Drug Deliv 23:3072–81. 26953869 10.3109/10717544.2016.1149744

[CIT0080] Meng J, Sturgis TF, Youan B-BC. (2011). Engineering tenofovir loaded chitosan nanoparticles to maximize microbicide mucoadhesion. Eur J Pharm Sci 44:57–67. 21704704 10.1016/j.ejps.2011.06.007 PMC3375325

[CIT0081] Mir M, Ishtiaq S, Rabia S, et al. (2017). Nanotechnology: from *in vivo* imaging system to controlled drug delivery. Nanoscale Res Lett 12:500. 28819800 10.1186/s11671-017-2249-8 PMC5560318

[CIT0082] Misra A, Hickey AJ, Rossi C, et al. (2011). Inhaled drug therapy for treatment of tuberculosis. Tuberculosis 91:71–81. 20875771 10.1016/j.tube.2010.08.009

[CIT0083] Mujoriya RZ, Bodla R. (2011). Niosomes–challenge in preparation for pharmaceutical scientist. Int J App Pharm 3:11–15.

[CIT0084] Mustapha O, Din FU, Kim DW, et al. (2016). Novel piroxicam-loaded nanospheres generated by the electrospraying technique: physicochemical characterisation and oral bioavailability evaluation. J Microencapsul 33:323–30. 27188242 10.1080/02652048.2016.1185475

[CIT0085] Muzzalupo R. (2016). Niosomes and proniosomes for enhanced skin delivery. In: Percutaneous penetration enhancer’s chemical methods in penetration enhancement. New York: Springer, 147–60.

[CIT0086] Nagarwal RC, Kumar R, Pandit J. (2012). Chitosan coated sodium alginate–chitosan nanoparticles loaded with 5-FU for ocular delivery: *in vitro* characterization and *in vivo* study in rabbit eye. Eur J Pharm Sci 47:678–85. 22922098 10.1016/j.ejps.2012.08.008

[CIT0087] Nahar K, Gupta N, Gauvin R, et al. (2013). *In vitro*, *in vivo* and *ex vivo* models for studying particle deposition and drug absorption of inhaled pharmaceuticals. Eur J Pharm Sci 49:805–18. 23797056 10.1016/j.ejps.2013.06.004

[CIT0088] Najlah M, Hidayat K, Omer HK, et al. (2015). A facile approach to manufacturing non-ionic surfactant nanodipsersions using proniosome technology and high-pressure homogenization. J Liposome Res 25:32–7. 24963602 10.3109/08982104.2014.924140

[CIT0089] Nasr M. (2010). *In vitro* and *in vivo* evaluation of proniosomes containing celecoxib for oral administration. AAPS PharmSciTech 11:85–9. 20058106 10.1208/s12249-009-9364-5 PMC2850489

[CIT0090] Neves JD, Palmeira-de-Oliveira R, Palmeira-De-Oliveira A, et al. (2014). Vaginal mucosa and drug delivery. In: Mucoadhesive materials and drug delivery systems. Chichester: Wiley, 99–132.

[CIT0091] Ning M, Guo Y, Pan H, et al. (2005). Niosomes with sorbitan monoester as a carrier for vaginal delivery of insulin: studies in rats. Drug Deliv 12:399–407. 16253956 10.1080/10717540590968891

[CIT0092] Paecharoenchai O, Teng L, Yung BC, et al. (2013). Nonionic surfactant vesicles for delivery of RNAi therapeutics. Nanomedicine (Lond) 8:1865–73. 24156490 10.2217/nnm.13.155 PMC3971008

[CIT0093] Pathan IB, Setty CM. (2009). Chemical penetration enhancers for transdermal drug delivery systems. Trop J Pharm Res 8:2.

[CIT0094] Pham TT, Jaafar-Maalej C, Charcosset C, Fessi H. (2012). Liposome and niosome preparation using a membrane contactor for scale-up. Colloids Surf B 94:15–21. 10.1016/j.colsurfb.2011.12.036 22326648

[CIT0095] Rahman SA, Abdelmalak NS, Badawi A, et al. (2015). Formulation of tretinoin-loaded topical proniosomes for treatment of acne: *in-vitro* characterization, skin irritation test and comparative clinical study. Drug Deliv 22:731–9. 24670094 10.3109/10717544.2014.896428

[CIT0096] Rajabalaya R, Leen G, Chellian J, et al. (2016). Tolterodine tartrate proniosomal gel transdermal delivery for overactive bladder. Pharmaceutics 8:27. 10.3390/pharmaceutics8030027 PMC5039446 27589789

[CIT0097] Rajera R, Nagpal K, Singh SK, Mishra DN. (2011). Niosomes: a controlled and novel drug delivery system. Biol Pharm Bull 34:945–53. 21719996 10.1248/bpb.34.945

[CIT0098] Ramesh YV, Jawahar N, Jakki SL. (2013). Proniosomes: a novel nano vesicular transdermal drug delivery. J Pharm Sci Res 5:153–8.

[CIT0099] Rashid R, Kim DW, Abid Mehmood Yousaf OM, et al. (2015a). Comparative study on solid self-nanoemulsifying drug delivery and solid dispersion system for enhanced solubility and bioavailability of ezetimibe. Int J Nanomedicine 10:6147. 26491288 10.2147/IJN.S91216 PMC4598224

[CIT0100] Rashid R, Kim DW, Ud Din F, et al. (2015b). Effect of hydroxypropylcellulose and Tween 80 on physicochemical properties and bioavailability of ezetimibe-loaded solid dispersion. Carbohydr Polym 130:26–31. 26076597 10.1016/j.carbpol.2015.04.071

[CIT0101] Ruckmani K, Sankar V, Sivakumar M. (2010). Tissue distribution, pharmacokinetics and stability studies of zidovudine delivered by niosomes and proniosomes. J Biomed Nanotechnol 6:43–51. 20499831 10.1166/jbn.2010.1101

[CIT0102] Sahoo RK, Biswas N, Guha A, Kuotsu K. (2014). Maltodextrin based proniosomes of nateglinide: bioavailability assessment. Int J Biol Macromol 69:430–4. 24909314 10.1016/j.ijbiomac.2014.05.075

[CIT0103] Sankar V, Hearnden V, Hull K, et al. (2011). Local drug delivery for oral mucosal diseases: challenges and opportunities. Oral Dis 17:73–84. 21382140 10.1111/j.1601-0825.2011.01793.x

[CIT0104] Santos SS, Lorenzoni A, Ferreira LM, et al. (2013). Clotrimazole-loaded Eudragit^®^ RS100 nanocapsules: preparation, characterization and in vitro evaluation of antifungal activity against Candida species. Mater Sci Eng C 33:1389–94. 10.1016/j.msec.2012.12.040 23827586

[CIT0105] Santos T, Maia J, Agasse F, et al. (2012). Nanomedicine boosts neurogenesis: new strategies for brain repair. Integr Biol 4:973–81. 10.1039/c2ib20129a 22801448

[CIT0106] Shaker DS, Nasr M, Mostafa M. (2013). Bioavailability and hypocholesterolemic effect of proniosomal simvastatin for transdermal delivery. Int J Pharm Pharm Sci 5:344–51.

[CIT0107] Shehata TM, Abdallah MH, Ibrahim MM. (2015). Proniosomal oral tablets for controlled delivery and enhanced pharmacokinetic properties of acemetacin. AAPS PharmSciTech 16:375–83. 25319057 10.1208/s12249-014-0233-5 PMC4370976

[CIT0108] Shukr MH, Eltablawy NA. (2015). Development and optimization of novel controlled-release pioglitazone provesicular powders using 32 factorial design. Drug Deliv and Transl Res 5:51–62. 25787339 10.1007/s13346-014-0215-x

[CIT0109] Soliman SM, Abdelmalak NS, El-Gazayerly ON, Abdelaziz N. (2016). Novel non-ionic surfactant proniosomes for transdermal delivery of lacidipine: optimization using 23 factorial design and in vivo evaluation in rabbits. Drug Deliv 23:1608–22. 26758033 10.3109/10717544.2015.1132797

[CIT0110] Song S, Tian B, Chen F, et al. (2015). Potentials of proniosomes for improving the oral bioavailability of poorly water-soluble drugs. Drug Dev Ind Pharm 41:51–62. 24111828 10.3109/03639045.2013.845841

[CIT0111] Todoroff J, Vanbever R. (2011). Fate of nanomedicines in the lungs. Curr Opin Colloid Interface Sci 16:246–54.

[CIT0112] Tu Y, Sun D, Zhang J, et al. (2014). Preparation and characterisation of andrographolide niosomes and its anti-hepatocellular carcinoma activity. J Microencapsulation 31:307–16. 24124885 10.3109/02652048.2013.843727

[CIT0113] Ud Din F, Kim DW, Choi JY, et al. (2017). Irinotecan-loaded double-reversible thermogel with improved antitumor efficacy without initial burst effect and toxicity for intramuscular administration. Acta Biomater 54:239–48. 28285074 10.1016/j.actbio.2017.03.007

[CIT0114] Ud Din F, Mustapha O, Kim DW, et al. (2015a). Novel dual-reverse thermosensitive solid lipid nanoparticle-loaded hydrogel for rectal administration of flurbiprofen with improved bioavailability and reduced initial burst effect. Eur J Pharm Biopharm 94:64–72. 25979136 10.1016/j.ejpb.2015.04.019

[CIT0115] Ud Din F, Rashid R, Mustaph a, O, et al. (2015b). Development of a novel solid lipid nanoparticles-loaded dual-reverse thermosensitive nanomicelle for intramuscular administration with sustained release and reduced toxicity. RSC Adv 5:43687–94.

[CIT0116] Vanić Ž, Hurler J, Ferderber K, et al. (2014). Novel vaginal drug delivery system: deformable propylene glycol liposomes-in-hydrogel. J Liposome Res 24:27–36. 23931627 10.3109/08982104.2013.826242

[CIT0117] Vanić Ž, Škalko-Basnet N. (2013). Nanopharmaceuticals for improved topical vaginal therapy: can they deliver? Eur J Pharm Sci 50:29–41. 23684936 10.1016/j.ejps.2013.04.035

[CIT0118] Veerareddy PR, Bobbala SKR. (2013). Enhanced oral bioavailability of isradipine via proniosomal systems. Drug Dev Ind Pharm 39:909–17. 22998221 10.3109/03639045.2012.717945

[CIT0119] Verma P, Prajapati SK, Yadav R, et al. (2016). Single intravenous dose of novel flurbiprofen-loaded proniosome formulations provides prolonged systemic exposure and anti-inflammatory effect. Mol Pharm 13:3688–99. 27632682 10.1021/acs.molpharmaceut.6b00504

[CIT0120] Walve J, Rane B, Gujrathi N, et al. (2011). Proniosomes: a surrogated carrier for improved transdermal drug delivery system. Int J Res Ayurveda Pharm 2:743–50.

[CIT0121] Wen MM, Farid RM, Kassem AA. (2014). Nano-proniosomes enhancing the transdermal delivery of mefenamic acid. J Liposome Res 24:280–9. 24779560 10.3109/08982104.2014.911313

[CIT0122] Wohlrab J, Kreft B, Tamke B. (2011). Skin tolerability of transdermal patches. Expert Opin Drug Deliv 8:939–48. 21506903 10.1517/17425247.2011.574689

[CIT0123] Yadav JD, Kulkarni PR, Vaidya KA, Shelke GT. (2011). Niosomes: a review. J Pharm Res 4:632–6.

[CIT0124] Yang S, Chen Y, Ahmadie R, Ho EA. (2013). Advancements in the field of intravaginal siRNA delivery. J Control Release 167:29–39. 23298612 10.1016/j.jconrel.2012.12.023

[CIT0125] Yasam VR, Jakki SL, Natarajan J, Kuppusamy G. (2014). A review on novel vesicular drug delivery: proniosomes. Drug Deliv 21:243–9. 24128089 10.3109/10717544.2013.841783

[CIT0126] Yuksel N, Bayindir ZS, Aksakal E, Ozcelikay AT. (2016). *In situ* niosome forming maltodextrin proniosomes of candesartan cilexetil: *in vitro* and *in vivo* evaluations. Int J Biol Macromol 82:453–63. 26455402 10.1016/j.ijbiomac.2015.10.019

[CIT0127] Zaki Ahmad M, Akhter S, Mohsin N, et al. (2014). Transformation of curcumin from food additive to multifunctional medicine: nanotechnology bridging the gap. CDDT 11:197–213. 10.2174/1570163811666140616153436 24934264

[CIT0128] Zhou QT, Tang P, Leung SSY, et al. (2014). Emerging inhalation aerosol devices and strategies: where are we headed? Adv Drug Deliv Rev 75:3–17. 24732364 10.1016/j.addr.2014.03.006

[CIT0129] Zidan AS, Mokhtar M. (2011). Multivariate optimization of formulation variables influencing flurbiprofen proniosomes characteristics. J Pharm Sci 100:2212–21. 21259237 10.1002/jps.22453

